# Performance Evaluation of the BD SARS-CoV-2 Reagents for the BD MAX System

**DOI:** 10.1128/JCM.01019-21

**Published:** 2021-11-18

**Authors:** Karen Yanson, William Laviers, Lori Neely, Elizabeth Lockamy, Luis Carlos Castillo-Hernandez, Christopher Oldfied, Ronald Ackerman, Jamie Ackerman, Daniel A. Ortiz, Sixto Pacheco, Patricia J. Simner, Stephen Young, Erin McElvania, Charles K. Cooper

**Affiliations:** a Becton, Dickinson and Company, BD Life Sciences—Integrated Diagnostic Solutions, Sparks, Maryland, USA; b CTMD Research, Palm Springs, Florida, USA; c Fellows Research Alliance, Inc., Savannah, Georgia, USA; d Comprehensive Clinical Research, LLC, West Palm, Florida, USA; e William Beaumont Hospital, Royal Oak, Michigan, USA; f BioCollections Worldwide, Inc., Miami, Florida, USA; g Johns Hopkins University School of Medicine, Department of Pathology, Division of Medical Microbiology, Baltimore, Maryland, USA; h Tricore Reference Laboratory, Albuquerque, New Mexico, USA; i NorthShore University Health System, Department of Pathology and Laboratory Medicine, Evanston, Illinois, USA; UNC School of Medicine

**Keywords:** BD MAX, COVID-19, Hologic Aptima, RT-qPCR, SARS-CoV-2, agreement, nucleic acid amplification test

## Abstract

Nucleic acid amplification testing (NAAT) for SARS-CoV-2 is the standard approach for confirming COVID-19 cases. This study compared results between two emergency use authorization (EUA) NAATs, with two additional EUA NAATs utilized for discrepant testing. The limits of detection (LOD) for the BD SARS-CoV-2 reagents for the BD MAX system (MAX SARS-CoV-2 assay), the bioMérieux BioFire respiratory panel 2.1 (BioFire SARS-CoV-2 assay), the Roche cobas SARS-CoV-2 assay (cobas SARS-CoV-2 assay), and the Hologic Aptima SARS-CoV-2 assay Panther (Aptima SARS-CoV-2 assay) NAAT systems were determined using a total of 84 contrived nasopharyngeal specimens with 7 target levels for each comparator. The positive and negative percent agreement (PPA and NPA, respectively) of the MAX SARS-CoV-2 assay, compared to the Aptima SARS-CoV-2 assay, was evaluated in a postmarket clinical study utilizing 708 nasopharyngeal specimens collected from suspected COVID-19 cases. Discordant testing was achieved using the cobas and BioFire SARS-CoV-2 NAATs. In this study, the measured LOD for the MAX SARS-CoV-2 assay (251 copies/ml; 95% confidence interval [CI], 186 to 427) was comparable to the cobas SARS-CoV-2 assay (298 copies/ml; 95% CI, 225 to 509) and the BioFire SARS-CoV-2 assay (302 copies/ml; 95% CI, 219 to 565); the Aptima SARS-CoV-2 assay had an LOD of 612 copies/ml (95% CI, 474 to 918). The MAX SARS-CoV-2 assay had a PPA of 100% (95% CI, 97.3% to 100.0%) and an NPA of 96.7% (95% CI, 94.9% to 97.9%) compared to the Aptima SARS-CoV-2 assay. The clinical performance of the MAX SARS-CoV-2 assay agreed with another sensitive EUA assay.

## INTRODUCTION

Since December 2019, when a cluster of cases was first reported in Wuhan, China, the COVID-19 pandemic, caused by the SARS-CoV-2 virus, has been a major global public health crisis (1). As of early August 2021, more than 201 million cases and 4.27 million deaths have been identified worldwide, with more than 35.6 million cases and 615 thousand deaths in the United States alone (2). Rapid transmission and lack of treatment make it difficult to mitigate the pandemic (3). Isolating suspected patients and executing effective contact tracing is critical for managing the spread of the disease (4). Diagnosis of COVID-19, through accurate detection of SARS-CoV-2 is the first step in guiding health care providers to triage patients, determine the treatment plan, and quarantine suspected contacts.

In response to the COVID-19 pandemic, the World Health Organization (WHO) and the U.S. Food and Drug Administration (FDA) issued emergency use authorization (EUA) for the development of *in vitro* diagnostic assays (5, 6). Several commercial manufacturers have developed nucleic acid amplification tests (NAATs) for SARS-CoV-2 testing. NAATs are a highly sensitive and specific method of SARS-CoV-2 detection for the diagnosis of COVID-19 in upper respiratory specimens collected through swab sampling such as nasopharyngeal, oropharyngeal, midturbinate nasal, or anterior nasal swabs (7–9). Implementation of NAAT testing on automated platforms has helped to ensure rapid turnaround times for patient results while maintaining a high level of performance for detection of SARS-CoV-2.

The BD SARS-CoV-2 reagents for the BD MAX system (MAX SARS-CoV-2 assay; Becton, Dickinson and Company, BD Life Sciences–Integrated Diagnostic Solutions, Sparks, MD, USA) uses real-time, reverse transcriptase quantitative PCR (RT-qPCR) and utilizes multiplexed primers and probes that are designed to amplify two unique regions of the SARS-CoV-2 nucleocapsid (N) gene, N1 and N2, and the human ribonuclease P (RNase P) gene. This assay received FDA EUA on 8 April 2020 (10). Due to an increase in false-positive result complaints (11, 12), an internal investigation was launched by the manufacturer. To enhance performance and reduce the potential for false-positive results, two modifications were made to the assay and authorized in September 2020 by the FDA: (i) an increase to the cutoff for the N2 channel and (ii) an improvement to the probe chemistry to reduce the background fluorescence. The performance evaluation described in this article utilized the modified assay and supported removal of the presumptive-positive limitation by the FDA in March 2021 (13).

The objective of this study was to assess the performance of the modified MAX SARS-CoV-2 assay for detection of SARS-CoV-2 in nasopharyngeal specimens collected consecutively from individuals suspected of COVID-19. The analytical sensitivity was first determined for the MAX SARS-CoV-2 assay and three other commercial SARS-CoV-2 NAAT assays. The clinical performance of the MAX SARS-CoV-2 assay was further examined by determining the positive percent and negative percent agreements (PPA and NPA, respectively). The utilization of multiple assays here facilitated comprehensive discordant testing in the absence of an established clinical reference standard for SARS-CoV-2 NAATs.

## MATERIALS AND METHODS

### Specimens and assays.

The first study compared the analytical sensitivity of the MAX SARS-CoV-2 assay, the bioMérieux BioFire respiratory panel 2.1 (BioFire SARS-CoV-2 assay; bioMérieux, BioFire Diagnostics, Salt Lake City, UT, USA), the Roche cobas SARS-CoV-2 assay (cobas SARS-CoV-2 assay; Roche Diagnostics, Indianapolis, IN, USA), and the Hologic Aptima SARS-CoV-2 assay Panther system (Aptima SARS-CoV-2 assay; Hologic, Marlborough, MA, USA). A total of 84 contrived nasopharyngeal specimens were prepared for each commercial assay. The heat-inactivated virus (ATCC, catalog [Cat.] no. VR-1986HK), quantified by the vendor using digital droplet PCR, was serially diluted in Tris-EDTA buffer and then added to simulated nasopharyngeal matrix prepared in universal viral transport medium (VTM; BD universal viral transport; BD Diagnostics, cat. no. 220220). The simulated nasopharyngeal matrix contains 175 mM sodium chloride (Fisher Scientific, cat. no. AM9759), 10 mM potassium chloride (Fisher Scientific, cat. no. AM9640G), 5 mM calcium chloride (Fisher Scientific, cat. no. BP9742), 0.75 mg/ml albumin (Sigma-Aldrich, cat. no. A1653), 1 mg/ml IgG antibodies (Sigma-Aldrich, cat. no. 56834), 0.02 mg/ml IgM antibodies (Sigma-Aldrich, cat. no. I8135), 2 mg/ml bovine mucin (Sigma-Aldrich, cat. no. M3895), and 6E4 cells/ml human lung epithelial cells (ATCC, cat. no. A549 CCL-185) prepared in universal transport medium. A total of six concentration levels (22, 67, 200, 600, 1,800, and 5,400 copies/ml) of contrived specimens were established to generate a panel for each assay. An additional negative-control level was also prepared for each panel.

The second study involved postmarket clinical testing using 1,376 specimens from four collection sites in the United States (see Table S1 in the supplemental material). The specimens included prospective as well as consecutively collected remnant nasopharyngeal swabs (BD Flexible Minitip flocked swab, cat. no. 220252) from symptomatic patients suspected of COVID-19 by their health care providers following the manufacturer’s instructions for use and maintained in VTM or Copan universal transport medium (UTM) (Copan Diagnostics). The choice of comparator method was based on feedback from the FDA on the post-EUA study design. There were 64 specimens that had an Aptima SARS-CoV-2 result but were not tested on MAX SARS-CoV-2. There were also 288 specimens that were enrolled but were not tested on either Aptima SARS-CoV-2 or MAX SARS-CoV-2 since the positive target goal was attained. Overall, 708 specimens were utilized for paired testing and analysis. Demographic information for compliant specimens with reportable results included 59.6% (422/708) female and 40.4% (286/708) male. Age ranges included 4.2% (30/708) <18 years old, 79.7% (564/708) 18 to 64 years old, 15.8% (112/708) ≥65 years old, and 0.3% (2/708) unknown. The study protocol was approved by the Advarra Institutional Review Board, and deidentified specimens from collection sites were used for testing. Written, informed consent was obtained prior to any trial-related procedures. This study was conducted according to the principles set forth by the Declaration of Helsinki and good clinical practice.

### Data analysis.

The analytical sensitivity values for the four assays were determined by calculating the limit of detection (LOD) using probit regression analysis. The point estimate for LOD is the lowest detectable concentration of SARS-CoV-2 at which approximately 95% of all (true positive) replicates test positive. A goodness-of-fit test was performed using Pearson and deviance correlation methods. Only data following normality or having at least two functional data points from a comparator yielded an appropriate statistical fit. Log_10_ transformation of the measurements was also performed for analyses to improve statistical fit.

For the postmarket clinical study, the Aptima SARS-CoV-2 assay was chosen as the reference for evaluating the performance of MAX SARS-CoV-2 based on consultation by the FDA. The primary outcome measures were PPA and NPA point estimates (with calculated 95% confidence intervals [95% CI] using the Wilson score method) for the MAX SARS-CoV-2 assay compared to the Aptima SARS-CoV-2. Cohen’s kappa coefficient was utilized to gauge the agreement between two raters (reference versus index test) to classify results into mutually exclusive categories: *Κ* = (*P_o_*^−^*^P^e*)/1 − *P_e_* (<0, 0, and >0, respectively, indicate agreements of worse than, no better or worse than, and better than that expected by chance). Acceptance criteria for the MAX SARS-CoV-2 assay for FDA-EUA clearance for SARS-CoV-2 were ≥95% for both PPA and NPA (6). Only compliant and reportable results for both MAX SARS-CoV-2 and comparator assays were included. This article was prepared according to Standards for Reporting of Diagnostic Accuracy Studies (STARD) guidelines for diagnostic accuracy studies reporting (14). Data will be made publicly available upon publication and upon request.

## RESULTS

The MAX SARS-CoV-2 assay was subjected to a series of validations to determine the impact (if any) on analytical sensitivity and specificity resulting from the cutoff change on the N2 channel and the modification to the probe chemistry (10). As shown in Table 1, the LOD of the MAX SARS-CoV-2 assay was obtained and compared to three other commercially available SARS-CoV-2 NAATs; specifically, the BioFire SARS-CoV-2, the cobas SARS-CoV-2, and the Aptima SARS-CoV-2 assays. From a total of 84 contrived nasopharyngeal specimens with seven target levels, the MAX SARS-CoV-2 assay had the lowest LOD (251 copies/ml; 95% CI, 186 to 427) but was comparable to the cobas SARS-CoV-2 (298 copies/ml for target 1; 95% CI, 225 to 509) and BioFire SARS-CoV-2 (302 copies/ml; 95% CI, 219 to 565) assays. The Aptima SARS-CoV-2 assay showed the highest LOD (612 copies/ml; 95% CI, 474 to 918) but was within a 2-fold concentration range of the other assays.

**TABLE 1:** Limit of detection (LOD) of MAX SARS-CoV-2, BioFire SARS-CoV-2, cobas SARS-CoV-2, and Aptima SARS-CoV-2^*a*^

UVT copies/ml	Data for MAX SARS-CoV-2				Data for BioFire SARS-CoV-2		Data for cobas SARS-CoV-2			Data for Aptima SARS-CoV-2	
	*N*	SARS-CoV-2 pos	N1 pos	N2 pos	*N*	SARS-CoV-2 pos	*N*	Target 1 pos	Target 2 pos	*N*	SARS-CoV-2 pos
0	12	0 (0%)	0 (0%)	0 (0%)	12	0 (0%)	12	0 (0%)	0 (0%)	12	0 (0%)
22	12	3 (25%)	1 (8%)	3 (25%)	12	1 (8%)	12	0 (0%)	0 (0%)	12	0 (0%)
67	12	4 (33%)	4 (33%)	2 (17%)	12	6 (50%)	12	3 (25%)	8 (67%)	12	1 (8%)
200	12	10 (83%)	9 (75%)	10 (83%)	12	8 (67%)	11	7 (64%)	11 (100%)	12	4 (33%)
600	12	12 (100%)	12 (100%)	11 (92%)	12	12 (100%)	12	12 (100%)	12 (100%)	12	11 (92%)
1,800	12	12 (100%)	12 (100%)	12 (100%)	NT	NT (NA)	12	12 (100%)	12 (100%)	12	12 (100%)
5,400	12	12 (100%)	12 (100%)	12 (100%)	NT	NT (NA)	12	12 (100%)	12 (100%)	12	12 (100%)
LOD (log_10_ transformed) (95% CI)^*b*^		398 (224–2,239)	427 (240–1,660)	741 (353–3,631)		490 (257–2,138)		447 (263–1,622)	83 (NA)^*d*^		813 (490–2,692)
LOD (copies/ml) (95% CI)^*c*^		251 (186–427)	271 (205–445)	519 (377–896)^*d*^		302 (219–565)		298 (235–509)	77 (NA)^*d*^		612 (474–918)

a Pos, positive; NT, not tested; NA, not applicable.

b LOD values are from log_10_-transformed data of the same measurement value used during linear analysis (see footnote c). For example, the value 398 copies/ml was converted back from a log_10_-transformed value of 2.6. Log_10_-transformed analysis provided an improved statistical fit.

c LOD values are from linear data analysis.

d Poor statistical fit.

In the clinical evaluation study, a total of 708 specimens tested were included for paired analysis. A single target detection is sufficient to call a positive for MAX. Among the 708 analyzed samples, 157 were positive by the MAX SARS-CoV-2 assay, with 80.9% (127/157) of positive specimens receiving a threshold cycle (*C_T_*) score of ≤30 and 45.9% (72/157) receiving a *C_T_* score of ≤20 (Table S2). Compared to the Aptima SARS-CoV-2 assay, 138 were positive by both MAX SARS-CoV-2 and Aptima SARS-CoV-2 assays, while 551 tested negative by both assays, and 19 samples were discrepant. Therefore, MAX SARS-CoV-2 testing resulted in a PPA of 100% (95% CI, 97.3% to 100.0%) and an NPA of 96.7% (95% CI, 94.9% to 97.9%) compared to the Aptima SARS-CoV-2 assay (Table 2). Discordant results were observed from 19 specimens that were positive with the MAX SARS-CoV-2 assay but negative by the Aptima SARS-CoV-2 assay (Table 3). Among these, 5 specimens were N1 positive/N2 positive, 11 specimens were N1 positive/N2 negative, and 3 specimens were N1 negative/N2 positive. The BioFire SARS-CoV-2 and the cobas SARS-CoV-2 assays were utilized for discrepancy testing. Of the 19 discordant specimens, 4 tested positive by the cobas SARS-CoV-2 assay and 5 were positive by the BioFire SARS-CoV-2 assay. One of the specimens did not generate a reportable result from either the cobas SARS-CoV-2 assay or the BioFire SARS-CoV-2 assay. Of the 19 discordant specimens, 5 did not have sufficient volume for BioFire SARS-CoV-2 testing and 5 did not yield valid results from the cobas SARS-CoV-2 assay due to a low volume error. Overall, 7 of 19 MAX SARS-CoV-2-positive specimens were also positive in discordant testing. Further analysis revealed that only one of the specimen results corresponded to the MAX SARS-CoV-2 *C_T_* values of <30 (specimen ID no. 9 in Table 3); the other results were either at, or close to, the LOD for the MAX SARS-CoV-2 assay.

**TABLE 2:** Performance of the MAX SARS-CoV-2 assay for detection of SARS-CoV-2 compared to the reference^*a*^

Performance value	SARS-CoV-2
PPA	100% (95% CI, 97.3% to 100%)
NPA	96.7% (95% CI, 94.9% to 97.9%)
MAX (+)/ref (+)	138
MAX (+)/ref (−)	19
MAX (−)/ref (+)	0
MAX (−)/ref (−)	551
Kappa	0.9187

a PPA, positive percent agreement; NPA, negative percent agreement. The reference method was the Aptima SARS-CoV-2 assay.

**TABLE 3:** List of MAX SARS-CoV-2 (+)/Aptima SARS-CoV-2 (−) specimens^*a*^

Specimen ID	MAX N1 *C_T_*	MAX N2 *C_T_*	MAX RNaseP *C_T_*	cobas target 1	cobas target 2	BioFire
1	37.1	−1	24.7	+ (33.86)	+ (35.25)	+
2	34.9	33.2	22.5	−	+ (36.99)	+
3	34.9	−1	21	−	+ (35.27)	QNS
4	37.9	−1	23.4	−	+ (37.02)	QNS
5	−1	35	22.3	INV	INV	+
6	35.3	34.4	26.4	INV	INV	+
7	37.5	−1	24	−	−	+
8	40.2	−1	22.8	−	−	QNS
9^*b*^	22.9	24.5	19.7	−	−	QNS
10	38.8	−1	22	INV	INV	−
11	37.4	−1	26.2	INV	INV	−
12	33.3	33.2	28.1	−	−	−
13	39.4	−1	25.1	−	−	−
14	35	−1	25	−	−	−
15	33.8	−1	26.8	−	−	−
16	31.2	−1	29.1	−	−	−
17	−1	35.9	26.8	−	−	−
18	−1	35.5	19.4	−	−	−
19	32.9	33.6	22.5	INV	INV	QNS

a INV, invalid value; QNS, quantity not sufficient for testing.

b With the exception of sample 9, all discordant samples exhibit *C_T_* values at or near the limit of detection on the MAX SARS-CoV-2 system.

## DISCUSSION

On 10 March 2021, the FDA provided EUA for the modified MAX SARS-CoV-2 RT-qPCR assay, which eliminated the condition of follow-up testing for a MAX SARS-CoV-2 assay presumptive positive (13). Here, the LOD values for the MAX and Aptima SARS-CoV-2 assays had LOD values of 251 and 612 copies/ml, respectively. The clinical study, incorporating 708 real-world specimens, resulted in 100% PPA and 96.7% NPA performance values for MAX SARS-CoV-2 assay compared to the reference (Aptima) assay and met FDA EUA acceptance criteria of greater than 95% for PPA and NPA. The updated BD MAX SARS-CoV-2 assay evaluated here is the current, commercially available EUA version of the test.

In this study, the MAX SARS-CoV-2 assay was compared to the Aptima SARS-CoV-2 assay with BioFire and cobas SARS-CoV-2 testing used to resolve discrepant results. A total of 19 discordant results occurred from 708 paired specimens. Upon discordant method testing, five specimens were positive by at least one additional NAAT, and two additional specimens were positive for cobas SARS-CoV-2 Target 2 (sarbecovirus). Several factors, including primer design, type of polymerase employed, reaction conditions, and template purity, could impact the analytical sensitivity of NAATs (15). In the absence of a consensus clinical reference standard for detection of SARS-CoV-2 via nucleic acid amplification, it is difficult to adjudicate, with certainty, which of the remaining 14 positive specimens by the MAX SARS-CoV-2 assay were truly positive. In addition, only 10 discordant specimens were tested with both cobas SARS-CoV-2 and BioFire SARS-CoV-2, due to specimen volume availability.

The LOD study we performed provides a direct comparison of four assays, and the results revealed LOD point estimates with 95% confidence intervals that largely overlap between the NAATs. Although we found the LODs of MAX, cobas, and BioFire SARS-CoV-2 assays to be similar, with Aptima SARS-CoV-2 slightly higher, the LOD values for some of the assays reported here do differ from values in previous publications and EUA submissions. The Aptima assay, which utilizes transcription-mediated amplification (TMA), has been previously shown to have an LOD of 150 copies/ml (16), and some studies even showed it to be more sensitive than RT-qPCR (17). The cobas LOD was also shown to be less than 100 copies/ml in two different studies (18, 19), while results for BioFire LOD testing from this study appear to be comparable to what others have seen (20, 21). There is lot-to-lot variability in the quantification of purchased reference standards used to make the contrived specimens, which may result in different LOD values across studies.

Additional factors may impact the LOD of SARS-CoV-2 NAATs. The genomic organization of SARS-CoV-2 viral RNA includes the open reading frame 1 (ORF1) and several regions encoding structural proteins, such as spike protein (S), envelope (E), membrane protein (M), and nucleocapsid (N). Among these structural genes, the N gene expresses the most abundant subgenomic mRNA transcript and could provide a higher starting amount of template, giving the MAX SARS-CoV-2 assay a lower apparent LOD (22). In addition, although the minimum required volume of UTM was taken for each assay, the largest (minimum) volume taken for the four assays was for the MAX SARS-CoV-2 assay (750 μl). The larger input volume associated with the MAX SARS-CoV-2 assay could have facilitated a lower LOD by providing more template from which to amplify (10, 23–25).

Accurate detection of SARS-CoV-2 variants by current NAATs continues to be a concern as the virus mutates (26, 27). If genetic mutations occur within the target regions of the SARS-CoV-2 primer, proper primer and probe binding may be affected and fail to detect the presence of the virus (28). To date, multiple mutations have been mapped out, and most of them are identified at the ORF1 region, followed by S, N, M, and E genes (29). The specificity of the N1 and N2 primer sets used by the MAX SARS-CoV-2 assay was determined using an *in silico* approach to compare with all 1,548,814 available SARS-CoV-2 sequences in the GISAID database as of 24 June 2021. Alignments against the N gene showed that both N1 and N2 primer/probe sets are a perfect match to 93.8% of sequences in the database, 96.8% of the sequences are a perfect match to the N1 primer set region, and 97.0% are a perfect match to the N2 primer set region. In total, 99.9% are a perfect match to either the N1 or the N2 region primer set. Additionally, N1 and N2 primers showed no significant combined homologies with human genome regions, other coronaviruses, or human microflora that would predict potential false-positive NAAT results (10, 23–25). Future work is required to determine the impact of assay design for providing comprehensive detection of the major SARS-CoV-2 variants.

Although the NAAT testing method is a highly sensitive and favorable approach to detect SARS-CoV-2 virus, several factors mentioned above could potentially affect the detection ability of the system, and 100% agreement between assays should not be expected. In conclusion, we found that the updated MAX SARS-CoV-2 assay has an LOD that is comparable to three other commercially available NAATs. Testing of clinical specimens showed that the MAX assay was highly sensitive and specific for detection of SARS-CoV-2 in patients with signs and symptoms of COVID-19 infection.
