# Supplementary material for: Performance Evaluation of the BD SARS-CoV-2 Reagents for the BD MAX System
Source: J Clin Microbiol. 2021 Nov 18;59(12):e01019-21. doi: 10.1128/JCM.01019-21 (PMC8601249; doi:10.1128/JCM.01019-21)
Supplement: Supplemental file 1 — Table S1. Download jcm.01019-21-s0001.pdf, PDF file, 0.09 MB [file jcm.01019-21-s0001.pdf]

**Supplemental Table 1.** Enrollment and compliance summary.

| Collection Site | Total # Enrolled | # of Compliant Specimens | Reportable Aptima and MAX Results[a] |
|---|---|---|---|
| A | 248 | 244 | 110 |
| B | 450 | 445 | 237 |
| C | 150 | 141 | 130 |
| D | 528 | 231 | 231 |
| Overall | 1376 | 1061 | 708 |

[a]There were 64 specimens that had an Aptima SARS-CoV-2 result but were not tested on MAX SARS-CoV-2. There were also 288 specimens that were enrolled but were not tested on either Aptima SARS-CoV-2 or MAX SARS-CoV-2 since the positive target goal was reached.
