# Supplementary material for: Performance Evaluation of the BD SARS-CoV-2 Reagents for the BD MAX System
Source: J Clin Microbiol. 2021 Nov 18;59(12):e01019-21. doi: 10.1128/JCM.01019-21 (PMC8601249; doi:10.1128/JCM.01019-21)
Supplement: Supplemental file 2 — Table S2. Download jcm.01019-21-s0002.pdf, PDF file, 0.1 MB [file jcm.01019-21-s0002.pdf]

Supplemental Table 2. The number of positive specimens tested by MAX-SARS-CoV-2 and stratified by Ct score[a].

Ct Score	Number of MAX SARS-CoV-2 positive samples
Ct ≤20	72
Ct >20, ≤30	55
Ct >30	30
Total	157

[a]Ct is stratified based on the minimum of the Ct for N1 and N2 if both channels were positive. Ct for the positive channel was taken if only one channel was positive.
